# Large measles outbreak introduced by asylum seekers and spread among the insufficiently vaccinated resident population, Berlin, October 2014 to August 2015

**DOI:** 10.2807/1560-7917.ES.2017.22.34.30599

**Published:** 2017-08-24

**Authors:** Dirk Werber, Alexandra Hoffmann, Sabine Santibanez, Annette Mankertz, Daniel Sagebiel

**Affiliations:** 1 State Office for Health and Social Affairs, Berlin, Germany; 2 These authors contributed equally to this work; 3 Postgraduate Training for Applied Epidemiology (PAE), Robert Koch Institute, Berlin, Germany; 4 European Programme for Intervention Epidemiology Training (EPIET), European Centre for Disease Prevention and Control (ECDC), Stockholm, Sweden; 5 National Reference Center for Measles, Mumps, Rubella, Robert Koch Institute, Berlin, Germany

**Keywords:** measles, vaccine-preventable diseases, disease outbreaks, asylum seeker, surveillance, post-exposure prophylaxis

## Abstract

The largest measles outbreak in Berlin since 2001 occurred from October 2014 to August 2015. Overall, 1,344 cases were ascertained, 86% (with available information) unvaccinated, including 146 (12%) asylum seekers. Median age was 17 years (interquartile range: 4–29 years), 26% were hospitalised and a 1-year-old child died. Measles virus genotyping uniformly revealed the variant ‘D8-Rostov-Don’ and descendants. The virus was likely introduced by and initially spread among asylum seekers before affecting Berlin’s resident population. Among Berlin residents, the highest incidence was in children aged < 2 years, yet most cases (52%) were adults. Post-exposure vaccinations in homes for asylum seekers, not always conducted, occurred later (median: 7.5 days) than the recommended 72 hours after onset of the first case and reached only half of potential contacts. Asylum seekers should not only have non-discriminatory, equitable access to vaccination, they also need to be offered measles vaccination in a timely fashion, i.e. immediately upon arrival in the receiving country. Supplementary immunisation activities targeting the resident population, particularly adults, are urgently needed in Berlin.

## Introduction

Measles is a highly communicable viral disease causing substantial morbidity and mortality globally, mostly in low-income countries [1]. Vaccination can safely and effectively prevent measles disease and measles virus (MV)-induced immunosuppression, thereby also preventing all-cause secondary infectious diseases [2]. The World Health Organization (WHO) has targeted measles and rubella for Regional elimination, and Germany has committed to this goal [3]. The key strategy for elimination is to achieve and sustain a population coverage of ≥ 95% with two doses of a MV-containing vaccine [4,5]. Thus far, elimination has only been reached in the Americas [6,7]. The WHO European Region failed to achieve the target date for elimination in 2015 [8].

In Germany, immunisation is voluntary and the German Standing Committee on Vaccination (STIKO) recommends routine administration of two doses of measles vaccine, at 11–14 and 15–23 months of age [9]. Although vaccine coverage for measles has increased substantially in children at school entry (5- to 6-year-olds) in the last decade, it is still below the 95% target (93% in Germany for two doses of measles in 2014; 92% in Berlin) [10]. Since 2010, STIKO has additionally recommended measles vaccination for adults born after 1970 with incomplete or unknown vaccination status [11].

The European Union has recently experienced a very large influx of asylum seekers with more than 600,000 and 1.3 million registered first-time applicants in 2014 and 2015, respectively [12]; Germany had the largest number of applicants. In Berlin, a city with 3.5 million inhabitants [13], 12,079 asylum seekers (12% from Bosnia and Herzegovina) were registered in 2014. This number more than tripled in 2015 (n = 44,615), with a steep increase in the second half of the year. Refugees and asylum seekers (both hereafter referred to as ‘asylum seekers’) should have non-discriminatory, equitable access to healthcare services, including vaccines, irrespective of their legal status [14-17]. Their right to receive vaccinations in Germany is legally anchored in the Asylum Seekers' Benefit Act [18]. Asylum seekers are accommodated in initial reception centres or collective accommodation centres (both hereafter referred to as ‘asylum seeker homes’) upon arrival in Germany. The national recommendation for post-exposure intervention in any community home, including asylum seeker homes, is to vaccinate all persons older than 9 months of age within 72 h after contact with a measles case [11].

Measles has been notifiable in Germany since 2001. From 2004 to 2013, the annual incidence per million population ranged from 1.5 to 28 without a discernible secular downward trend. During that time, the annual measles incidence was highest in Berlin, driven, in part, by a large outbreak with almost 500 reported cases in 2013. In October 2014, measles cases started to accumulate again in Berlin. Initially, most cases were in asylum seekers. We enhanced epidemiological surveillance of measles, evaluated post-exposure vaccination in homes for asylum seekers, and performed molecular surveillance of MV circulation. This report describes the epidemiological and molecular characteristics of this outbreak.

## Methods

### Data sources

#### Notification database

In Germany, clinical suspicion or diagnosis of measles, measles-related death and laboratory detection of measles infection are notifiable to the Local Public Health Authority (LPHA). The surveillance case definition requires that the patient have fever, maculopapular rash and one of the following: cough, coryza, Koplik spots or conjunctivitis, or an epidemiological link to a person with laboratory-confirmed measles infection. Laboratory confirmation was defined as detecting MV nucleic acid by PCR, or MV-specific IgM antibodies or a significant increase in anti-MV IgG. Cases were transmitted to the State Office for Health and Social Affairs (SOHSA) and from there to the federal level public health institute.

#### Additional case information

We enhanced epidemiological surveillance of measles cases by requesting that LPHAs systematically collect additional information, including residency status (asylum seeker (Y/N)), nationality, whereabouts in the two weeks before symptom onset and duration of stay in Germany. Information was recorded in a specifically designed Excel sheet and sent to SOHSA.

#### Molecular data on MV

The National Reference Centre for Measles, Mumps, Rubella (NRC MMR) at the Robert Koch Institute, Berlin, determined the MV genotype in all clinical samples confirmed by detection of viral RNA that were collected in Berlin during the outbreak period. The ‘distinct sequence identifier’ representing each MV sequence variant was determined and compared using the global WHO Measles Nucleotide Surveillance (MeaNS) database [19]. The phylogenetic tree was constructed using the neighbour-joining algorithm and the p-distance method as implemented in MEGA 7 [20].

For measles cases, we merged information from the notification database with additional case information and molecular data on MV by using the case identifier of the notification system or by date of onset, age, and sex (for molecular data from the NRC MMR).

### Epidemiological investigation

We defined an outbreak case as illness in a person notified with measles in Berlin from 6 October 2014 (week 41) until 30 August 2015 (week 35), if the illness fulfilled the surveillance case definition and was not imported (except for the index patient), and the isolate, if genotyped, belonged to genotype D8. A case was considered to be imported if the patient was abroad during the 7 to 18 days before symptom onset.

We conducted a descriptive analysis of case characteristics by calculating frequencies and proportions or median values and interquartile ranges (IQR) as appropriate. Attack rates per million population, by age group and district, were computed for Berlin resident cases using 2013 population data from the Statistical Office for Berlin-Brandenburg.

### Evaluation of post-exposure vaccinations in asylum seeker homes

We evaluated timeliness and completeness of post-exposure vaccinations in asylum seeker homes between October 2014 and February 2015. Date of symptom onset and notification of the first case was extracted from the notification system, details on the intervention (e.g. date, number of (eligible) asylum seekers vaccinated/registered) were collected by LPHAs and collated by the SOHSA. We computed median values and IQR of time intervals characterising the timeliness of the intervention. Completeness was assessed only in post-exposure vaccinations targeting all asylum seekers, which excluded homes that had closed living units and interventions restricted to specific vulnerable subgroups, e.g. children or husbands of pregnant women. It was computed as the number of vaccinated asylum seekers divided by the total number of (eligible) registered asylum seekers. Persons older than 9 months of age who were not pregnant and had no clinical signs compatible with measles and no written documentation of two measles vaccinations were eligible.

### Molecular investigation

Suspected measles cases were confirmed in private or hospital laboratories or in the NRC MMR using tests for detection of anti-MV IgM in serum. Alternatively, MV RNA was detected in throat swabs, oral fluid or urine by an accredited PCR test conducted in the NRC MMR. Detected MV was genotyped by sequencing the 450 nucleotides (nt) coding for the C-terminal 150 amino acids of the nucleoprotein and phylogenetic analysis as recommended by the WHO [21]. Representative MV sequence data were submitted to the MeaNS database and to GenBank [19].

## Results

### Description of the outbreak

Of 1,359 cases notified during the outbreak period, 15 were considered unrelated to the outbreak; six because of genotypes other than D8 and nine because infection was considered imported, among them three asylum seekers (all from Bosnia and Herzegovina). Thus, 1,344 outbreak cases were ascertained, of which 943 (70%) were laboratory-confirmed (Table).

**Table:** Case characteristics in a large outbreak of measles in Berlin, October 2014–August 2015 (n = 1,344)

Characteristics	Berlin resident		Asylum seeker		Unknown		Total	
	n	%	n	%	n	%	n	%
**Number of cases**	1,101	100	146	100	97	100	1,344	100
**Male**	612	55.6	76	52.0	49	50.5	737	54.8
**Laboratory-confirmed**	777	70.6	99	67.8	67	69.1	943	70.2
**In clusters**	349	31.7	107	73.3	24	24.7	480	35.7
**Unvaccinated ^a^**	888	85.2	127	94.1	71	87.6	1,086	86.3
**Hospitalised ^b^**	265	24.1	35	24.0	45	46.9	345	25.7
**Death**	1	0.1	0	0	0	0	1	0.1
	**Median**	**IQR**	**Median**	**IQR**	**Median**	**IQR**	**Median**	**IQR**
**Age (years)**	18	5–30	5	2–18	21	9–29	17	4–29

IQR: interquartile range.

^a^ Information was available for 1,258 cases on vaccination status; 1,042 Berlin residents, 135 asylum seekers, 81 unknown.

^b^ Information was available for 1,342 on hospitalisation status; 1,100 Berlin residents, 146 asylum-seekers, 96 unknown.

Median age of case patients was 17 years (IQR: 4–29 years), 737 (55%) were male, 345 (26%) were hospitalised and a 1-year-old child died of measles. In almost two thirds of cases (64%, n = 864), no link to another measles case was recorded by the LPHAs. Of those with available information (n = 1,258), 86% (n = 1,086) were not vaccinated against measles, 101 (8%) were vaccinated once, 42 (3%) twice and one case three times (0.2%) before onset of illness (for 28 (2%) the number of vaccinations was not recorded).

**Figure 1:**
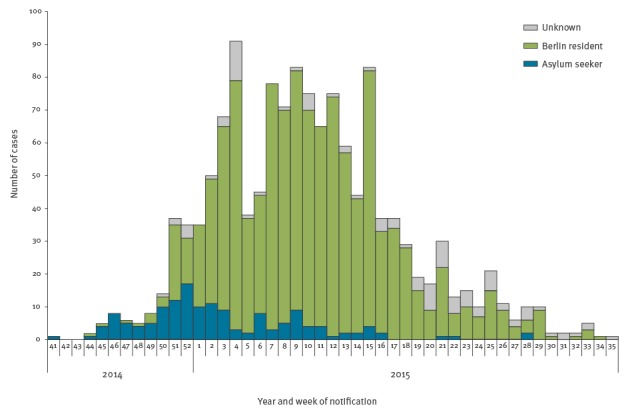
Cases by reporting week and residency status in a large outbreak of measles in Berlin, October 2014–August 2015 (n = 1,344)

The index case was a 5-year-old child who travelled with their family from Bosnia and Herzegovina by bus to Berlin (a ca 24 h drive) in early October 2014. The child had fever but no rash upon arrival. In the following months, cases occurred predominantly among asylum seekers (Figure 1).

During the outbreak, 146 (11%) cases occurred in asylum seekers (Table), most of them in children (median age: 5 years, IQR 2–18 years). Overall, 69 (47%) came from Bosnia and Herzegovina (in 2014: 41/65 cases, 63%), 41 (28%) from Serbia, eight (6%) from Syria, the remaining 20% came from 14 different countries. Measles cases occurred in 35 homes for asylum seekers, located in all Berlin districts, with a median of two cases per asylum seeker home (IQR: 1–5).

**Figure 2:**
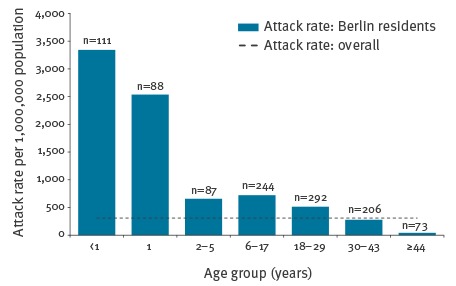
Attack rates per 1,000,000 by age group among the resident population in a large outbreak of measles in Berlin, October 2014–August 2015

By year’s end, cases started to accumulate in the Berlin resident population, with a peak in March 2015 (Figure 1). Cases among Berlin residents (n = 1,101) occurred in all districts, but the attack rate varied across the 12 districts of Berlin by a factor of almost 3.5 (highest in Neukölln: 546/1,000,000 population, lowest in Steglitz-Zehlendorf: 160/1,000,000 population (city-wide incidence: 309/1,000,000 population). Of the Berlin resident cases, almost one third (n = 349, 32%) were linked to other cases in 132 clusters (median number of cases: 2, IQR: 2–3), mostly to household members (253 cases in 106 clusters), meaning that no long transmission chains or sub-outbreaks were observed. Attack rate was highest among < 1 year olds (3,334/1,000,000, Figure 2) followed by 1 year-olds (2,538/1,000,000, which together accounted for 18% of Berlin’s resident cases. The majority of cases were in adults (n = 571, 52%), most of whom (498/571, 87%) were born after 1970.

### Evaluation of post-exposure vaccinations in asylum seeker homes

In the study period, cases were ascertained in 32 asylum seeker homes. Case-patients’ median age in clusters in asylum seeker homes was 4 years (IQR: 1–18.5 years). Of 32 asylum seeker homes, we received no detailed information for seven homes, and in a further seven homes no post-exposure intervention was performed for different reasons (e.g. lack of resources). In the remaining 18 homes, post-exposure vaccinations were conducted with a median time interval from symptom onset of the first case to vaccination of 7.5 days (IQR: 6–10 days); in three homes vaccination occurred within the recommended 72 h after detection of measles with no further cases notified during the following 18 days. In the remaining 15 homes, 16 cases were notified during this time period. Median time interval between (i) symptom onset of the first case and notification and (ii) notification and post-exposure vaccination was 4 days (IQR: 3–8 days) and 2 days (IQR: 0–6 days), respectively.

In the eight asylum seeker homes where vaccination was offered to all inhabitants and denominator data were available, 1,133/2,390 (47%) were reached. In five of these homes, eligibility criteria for vaccination were also recorded; there, 706/1,344 (53%) were reached.

### Molecular investigation

**Figure 3:**
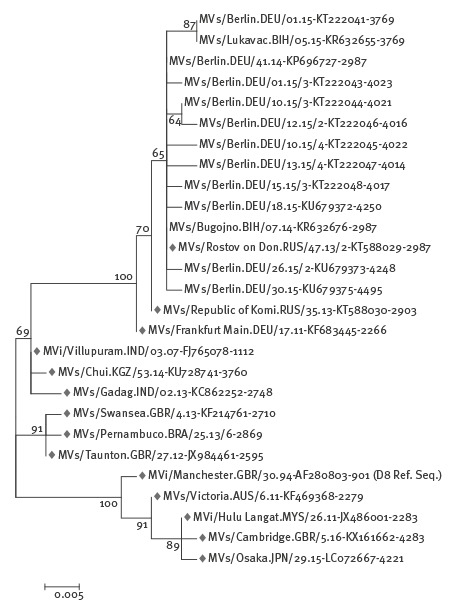
Phylogenetic relationship between the measles virus sequence variants detected in a large outbreak of measles in Berlin, August 2014–October 2015 (n = 351), a large outbreak in Bosnia and Herzegovina (2014–2015) and the World Health Organization Reference and Named Strains for measles virus genotype D8 (marked in grey)

During the outbreak period, the NRC received samples from 587 suspected cases. MV genome was detected in samples from 415 laboratory-confirmed cases and the MV genotype was determined for 359 cases of which 354 showed a wild-type virus. Of the 351 cases associated with genotype D8, 306 showed the predominant sequence variant Named Strain MVs/Rostov on Don.RUS/47.13/2, hereafter referred to as ‘D8-Rostov-Don’. This variant was detected in Berlin during the entire outbreak period and had previously been found during a large outbreak in Bosnia and Herzegovina (February 2014 to April 2015). Forty-five cases had variants deviating in the 450 nt region by 1–2 nt from D8-Rostov-Don (10 variants, Figure 3) [22]. One variant detected in Berlin from December 2014 to May 2015 (MVs/Berlin.DEU/01.15) was also found in the course of the aforementioned outbreak (MVs/Lukavac.BIH/05.15) [22].

The World Health Organization name, the GenBank accession number (if available) and the ‘distinct sequence identifier’ used in MeaNS are given for each sequence variant. The phylogenetic analysis is based on the 450-nt sequence encoding the C-terminus of the measles virus N protein. The tree was constructed using the Neighbour-Joining algorithm and the p-distance method as implemented in MEGA7 [20]. Bootstrap values (1,000 replicates) are shown next to the branches. The scale bar indicates a deviation of 5 nt per 1,000 nt sequence.

**Figure 4:**
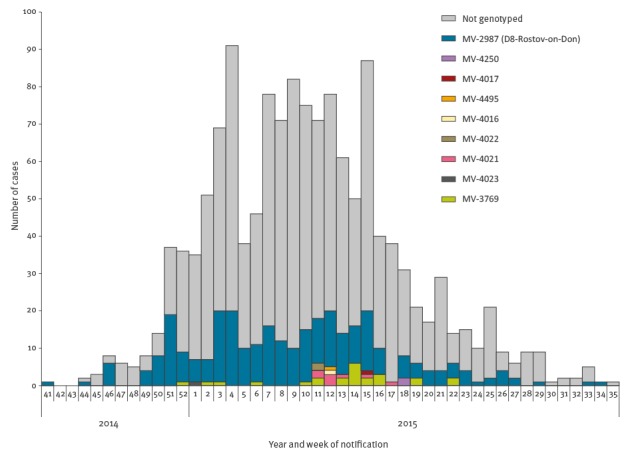
Measles virus sequence variants (given by the ‘distinct sequence identifier’) by reporting week of cases in a large outbreak in Berlin, October 2014–August 2015 (n = 322)

We were able to match 322 laboratory-confirmed cases with MV genotype D8 to notified outbreak cases (254 Berlin residents and 46 asylum seekers and in 22 with unknown residency status), of which 282 (88%) had the variant D8-Rostov-Don and 40 (12%) had a 1–2 nt deviating variant (Figure 4), 11 asylum seekers, 28 Berlin residents, one case with unknown residency status. None of these cases had a travel history during their period of infection. Of these 40 cases, 24 shared an identical variant (MVs/Lukavac.BIH/05.15). The first of these cases had a symptom onset on 20 December 2014 and the last on 21 May 2015.

## Discussion

In this large outbreak, MV was likely introduced by and initially spread among asylum seekers before affecting the resident population of Berlin, which ultimately accounted for the vast majority of cases. Among Berlin’s resident population, all age groups eligible for vaccination were affected, with the highest attack rate in children under 2 years of age. More than half of the cases were in adults, most of whom were born after 1970, the age group for which STIKO recommends measles vaccination if vaccination history is incomplete or unknown. Interventions of LPHAs in homes for asylum seekers were mostly outside the recommended 72 h time window, reached only half of the potential contacts and apparently did not halt the outbreak. Continuous genotyping throughout the outbreak period demonstrated almost endemic (i.e.  ≥ 12 months) transmission of the predominant variant D8-Rostov–Don in Berlin.

Several lines of evidence indicate that measles was introduced by and initially spread among asylum seekers: firstly, the index case occurred in an asylum seeker who had symptoms upon arrival in Berlin, after travelling from Bosnia and Herzegovina where a large measles outbreak was ongoing at that time [22]. Subsequent cases occurred predominantly among asylum seekers (often from Bosnia and Herzegovina) that acquired infection in Berlin. Secondly, genotyping data indicate sequence identity of MV isolated from the index case, the large majority of the following cases in Berlin, and cases of the concurrent outbreak in Bosnia and Herzegovina. Thirdly, despite of continuous monitoring of circulating MV variants, the main variant D8-Rostov-Don and closely related variants had never been found in Berlin before.

Population immunity in Berlin was too low to prevent city-wide measles spread across all age groups, equating to insufficient vaccine coverage. School-entry examinations document that coverage with two doses of a MV-containing vaccine has been consistently below 95% in Berlin, although there has been a strong increase over the past 15 years (from 21% in 1998–2001 to 92% in 2014) [10,23]. Age-dependent differences in vaccine coverage were observed in a national population survey from 2008 to 2011; coverage (1 dose) was lower in older age groups (80% in 18–29 year olds; 47% in 30–39 year olds) [24]. Taken together, population immunity likely decreases in Germany with increasing age, until age groups born before 1970.

The majority of cases were in adults. Of note, there is a clear shift towards adult measles cases in German notification data (from 11% in 2003 to 43% in 2013) [25], in the absence of a noticeable downward trend in measles notification rates over the past 10 years in Germany. Furthermore, under-reporting of measles in Germany is highest in adults [26]. Consequently, their involvement in this outbreak (and in measles epidemiology in Germany in general) can be assumed to be disproportionately underestimated and the difference in attack rates between adults and children in this outbreak is likely offset, at least partially. Interestingly, standard inquiries of cases in this outbreak by LPHAs revealed that adults tended to be ignorant of their vaccination status rather than sceptical of vaccines, suggesting they could be successfully targeted in catch-up campaigns (not yet conducted in Berlin or elsewhere in Germany) [27].

Post-exposure vaccination by LPHAs to prevent onward transmission in homes for asylum seekers were not always conducted and, where conducted, reached only half of the potential contacts. Additionally, most (83%) vaccination measures occurred after the recommended 72 h window, mainly because LPHAs were not notified in a timely fashion (vaccinating after 72 h may still be useful, e.g. to prevent tertiary cases). We did not systematically assess the reasons for delayed case notification. Anecdotally, not all cases sought medical attention immediately and sometimes the first notified case was initially misdiagnosed with a disease other than measles. Furthermore, the large number of asylum seekers in some homes often exceeded the response capacity of the LPHAs. Taken together, timely vaccination of all contacts proved challenging in these settings where LPHA often became aware of measles too late, potential contacts were often difficult to reach and large in number, and language barriers complicated intervention logistics. Of note, clusters occurred in many homes for asylum seekers (mostly involving children), but were small in size (median of two cases). This indicates a fairly high vaccination coverage among inhabitants as has been shown for migrating populations in other settings, an effect of post-exposure vaccinations, or both [28,29]. Targeting, or at least prioritising, vulnerable groups (e.g. children) in post-exposure vaccinations in (mass) asylum seeker homes should be considered, particularly in crisis situations.

It remains unclear why the outbreak became so large and long-lasting. Frequent introduction of the outbreak virus by different asylum seekers may seem an obvious explanation, but the limited available epidemiological and molecular information lends little support to this hypothesis. Only three measles cases among asylum seekers (all from Bosnia and Herzegovina) were considered imported. Yet, under-ascertainment of measles cases is likely, particularly at the start of the outbreak (e.g. there is a gap of > 3 weeks in onset dates between the index and subsequent cases). The identification of various outbreak variants is not necessarily indicative of imported MV. All variants were closely related to the main outbreak variant D8-Rostov-Don. Most were detected only once or over a short period, and their occurrence scattered around the (virus-number) peak of the outbreak, which is compatible with random mutations of the virus. Furthermore, detection of a variant probably descending from the outbreak main variant has previously been observed in a large outbreak, in which multiple importations were deemed unlikely [30]. In keeping with that pattern, none of the (matched) cases infected with an outbreak variant deviating from the main variant were imported. However, the predominant and the second most variant identified in Berlin were also detected in the outbreak in Bosnia and Herzegovina, indicating at least two virological links to the parallel outbreak. Notwithstanding, our molecular analysis was restricted to 450 nt of the MV N gene, and therefore does not allow the monitoring of sequence variation in other regions of the viral genome. Establishing whole genome sequencing in outbreak situations across Europe might provide more detailed information. More important, however, is a continuous monitoring of virus variants during the whole outbreak period, especially when considering increasing population movement in recent years.

Enhancing the surveillance of notified measles cases, including routinely collecting information on residency status, and MV genotyping, were pivotal in understanding the epidemiology of this outbreak. Since October 2015, residency status and accompanying information have been routinely collected on all notifiable diseases in Germany. Although the data need to be interpreted with caution, they indicate that most notified infectious diseases in asylum seekers are vaccine-preventable (predominantly varicella zoster infections), and most are acquired within Germany (data not shown). This underlines the vulnerability of this group for vaccine-preventable diseases.

No city-wide system for offering vaccinations to asylum seekers was in place during the outbreak period. Since September 2015, Berlin has offered immunisation with MV- (and polio-) containing vaccines, as recommended by WHO, the European Centre for Disease Prevention and Control and STIKO, to all asylum seekers in Berlin [31,32]. Coupled with a brief medical examination, this has become an integral part of the asylum seekers’ registration process since March 2016 in Berlin. Notwithstanding, there remains a continued risk of importation of MV as many residents travel and many travellers arrive. In 2014, 12.4 million people visited Berlin, of which 4.8 million came from abroad (one fifth of all foreign visitors to Germany) [33]. In conjunction with insufficient population immunity, eliminating measles in Berlin is likely to remain a distant prospect in the absence of supplementary immunisation activities.

## Conclusion

This outbreak exemplifies why, in addition to ethical and legal grounds, asylum seekers should be timely offered vaccination against measles: to protect them. In addition, catch-up campaigns to close immunisation gaps, particularly in adults, are urgently needed in Berlin’s resident population. Surveillance of infectious diseases should routinely collect information on residency status to be able to assess and quickly respond to infectious disease risks in asylum seekers. MV genotyping throughout the outbreak period demonstrated continuous circulation of variant D8-Rostov-Don for almost 11 months in Berlin.
